# Circ_0020014 mediates CTSB expression and participates in IL-1β-prompted chondrocyte injury via interacting with miR-24-3p

**DOI:** 10.1186/s13018-023-04370-8

**Published:** 2023-11-18

**Authors:** Chenpeng Zhang, Wenjun He

**Affiliations:** 1 Department of Spinal Surgery, Liyang People’s Hospital, Liyang, Jiangsu China; 2 Department of Osteoarthritis, Liyang People’s Hospital, Liyang, 213300 Jiangsu China

**Keywords:** Osteoarthritis, Circ_0020014, miR-24-3p, CTSB

## Abstract

**Background:**

Recent studies have shown that circRNAs are involved in the pathogenesis of osteoarthritis (OA) by affecting various fundamental cellular characteristics of chondrocytes. The purpose of this paper is to investigate the role and regulatory mechanism of hsa_circ_0020014 (circ_0020014) in chondrocytes of OA.

**Methods:**

The inflammatory cytokine interleukin 1 beta (IL-1β) was used to stimulate human chondrocytes. Cell viability, proliferation, and apoptosis were evaluated by 3-(4,5-dimethylthiazol-2-yl)-2,5-diphenyl tetrazolium bromide (MTT), 5-Ethynyl-2′-deoxyuridine (EdU), and flow cytometry assays. Several protein levels were determined by western blotting (WB). Levels of inflammatory cytokines and malondialdehyde (MDA) were determined by enzyme-linked immunosorbent assay (ELISA). Relative expression of circ_0020014 was estimated by real-time polymerase quantitative chain reaction (RT-qPCR). Bioinformatics prediction combined with dual-luciferase reporter, RIP and RNA pull-down assays were done to probe into the regulatory mechanism of circ_0020014.

**Results:**

Circ_0020014 was overexpressed in OA patient-derived articular cartilages and IL-1β-stimulated chondrocytes. Silencing of circ_0020014 lighted IL-1β-prompted chondrocyte proliferation repression, apoptosis, inflammation, and oxidative stress. Mechanically, circ_0020014 functioned as a miR-24-3p molecular sponge to regulate cathepsin B (CTSB) expression. Furthermore, miR-24-3p inhibition alleviated circ_0020014 knockdown-mediation repression of IL-1β-urged chondrocyte injury. In addition, CTSB overexpression whittled miR-24-3p upregulation-mediated suppression of IL-1β-urged chondrocyte injury.

**Conclusion:**

Our findings demonstrated that the circ_0020014/miR-24-3p/CTSB axis was associated with IL-1β-prompted chondrocyte injury, supporting the involvement of circ_0020014 in the OA pathogenesis.

**Supplementary Information:**

The online version contains supplementary material available at 10.1186/s13018-023-04370-8.

## Highlights

1. A significant upregulation of circ_0020014 was observed in OA patient-derived articular cartilages.
2. Inhibition of circ_0020014 lessened IL-1β-urged chondrocyte injury.
3. Circ_0020014 could interact with miR-24-3p to mediate CTSB expression.

**Supplementary Information:**

The online version contains supplementary material available at 10.1186/s13018-023-04370-8.

## Introduction

Osteoarthritis (OA) is a disabling age-related joint disease. Current treatments for OA aim to relieve symptoms, including temporary swelling and pain relief [1]. Articular cartilage degeneration, ligament and meniscus degeneration, osteophyte formation, and subchondral bone thickening are characteristics of OA [7]. Research on the pathogenesis of OA is indispensable at present.

Articular cartilage is an important structure that helps shock absorption and joint lubrication [12]. Loss of articular cartilage function caused by aging and trauma can lead to OA [17]. Articular cartilage is composed of chondrocytes that are critical for maintaining the integrity of the cartilage [22]. The inflammatory cytokine interleukin 1 beta (IL-1β) has been identified to trigger a range of cartilage-related catabolic effects [19]. Changes in chondrocyte survival induced by IL-1β may have important implications for the development of cartilage degeneration in OA [16]. Investigation regarding IL-1β-induced chondrocyte injury is helpful to explain the OA pathogenesis.

Circular RNAs (circRNAs) are endogenous RNA transcripts generated by the spliceosome-mediated head-to-tail joining of pre-mRNAs [5]. These circular RNAs easily escape from hydrolysis by numerous exonucleases such as RNase R due to the lack of free ends, and the reduced exonuclease sensitivity enables circRNAs to have significantly longer half-lives compared to linear RNAs [14]. They are expressed in a site-and stage-specific manner [21], and the disruption of circRNA expression is associated with human diseases [28]. Studies have shown that circRNAs exert their effects by sponging microRNAs (miRNAs), sequestering RNA-binding proteins (RBPs), or regulating RBP interactions [6]. A small subset of circRNAs has been validated to be involved in OA pathogenesis [15]. For instance, the interaction between circRNA-33186 and miR-127-5p was involved in the destabilized medial meniscus-induced destruction of articular cartilage in the distal femoral metaphysis of mouse knee joints [31]. CircRNA-SERPINE2 elevated ERG expression by adsorbing miR-1271-5p, resulting in alleviating cell apoptosis and promoting cell ECM anabolism in chondrocytes [23]. Hsa_circ_0020014 (circ_0020014), derived from the dual-specificity phosphatase 5 (DUSP5) gene, has been revealed as a potential biomarker for differential diagnosis of OA and Kashin-Beck disease [25]. At present, the action of circ_0020014 and its regulatory mechanism in IL-1β-induced chondrocyte dysfunction are still unclear.

In the research, we investigated the functional and molecular mechanisms of circ_0020014 in IL-1β-induced chondrocyte dysfunction. We believe that our study lays the cornerstone for future research on circRNAs as therapeutic targets for OA.

## Materials and methods

### Cell culture and stimulation

Human chondrocytes CHON-001 (Catalog # CRL-2846™, American Type Culture Collection, Manassas, VA, USA) were cultured in ATCC-derived Dulbecco’s Modified Eagle’s Medium (Catalog # 30-2002) supplemented with G-418 (0.1 mg/mL, Catalog # PB180125, Procell, Wuhan, China) and 10% heat-inactivated fetal bovine serum (Catalog # 10100147, Thermo, Waltham, MA, USA) in a suitable environment (5% carbon dioxide, 37 °C). For IL-1β stimulation, CHON-001 cells were stimulated with different doses of IL-1β (Sigma, St. Louis, MO, USA) (0, 5, 10, 15 ng/mL) for 24 h, and the dose of IL-1β at 10 ng/mL was utilized for functional analysis.

### Evaluation of cell viability

The 3-(4,5-dimethylthiazol-2-yl)-2,5-diphenyl tetrazolium bromide (MTT) assay was done with the Cell Proliferation and Cytotoxicity Assay Kit (Catalog # C0009S, Beyotime, Shanghai, China). In short, CHON-001 cells (5 × 10^3^) were stimulated with IL-1β and then incubated with the MTT solution (10 μL, 5 mg/mL) for 4 h. Absorbance was measured using the Microplate Reader (Bio-Rad, Hercules, CA, USA) at 570 nm after the purple crystals were completely dissolved with 100 μL of formazan solution.

### Detection of cell proliferation

CHON-001 cells were seeded in 6-well plates at a density of 3 × 10^5^ cells/well. After IL-1β stimulation, the proliferative capacity of the cells was tested in strict accordance with the instructions offered by the BeyoClick™ EdU Cell Proliferation Kit with Alexa Fluor 594 (Beyotime). EdU-positive cells were observed under an inverted microscope (Olympus, Tokyo, Japan) after nuclei were detected using 4′,6-diamidino-2-phenylindole staining solution (Beyotime).

### Analysis of cell apoptosis

Apoptosis was assessed in strict accordance with the protocol included in the Annexin V-FITC Apoptosis Detection Kit (Beyotime). After washing adherent cells with PBS, cells were digested with an EDTA-free trypsinization solution. Cells were collected by centrifugation, followed by re-suspending of approximately 1 × 10^5^ cells in 195 μL of Annexin V-FITC binding solution. The resuspended cells were incubated in the dark for 15 min after adding 5 μL of Annexin V-FITC and 10 μL of propidium iodide staining solution. Data were analyzed by FlowJo software (Tree Star, San Carlos, CA, USA) following detection with the flow cytometry (BD, San Jose, CA, USA).

### Western blotting (WB)

Harvested cells and clinical samples were lysed in RIPA buffer (Sigma) for total protein extraction. After detection of the protein concentration contained in the obtained supernatant, 10 μg of protein was separated by 10% sodium dodecyl sulfate–polyacrylamide gel electrophoresis and transferred to polyvinylidene fluoride (PVDF) membrane (Millipore), followed by blocking for 1 h in 5% skim milk. PVDF membranes were then incubated with rabbit anti-human Bax (ab32503, 1:1000, Abcam, Cambridge, MA, USA) antibody, rabbit anti-human Bcl-2 (ab182858, 1:2000, Abcam) antibody, rabbit anti-human CTSB (ab125067, 1:1000, Abcam) antibody, and rabbit anti-human GAPDH (ab181602, 1:10,000, Abcam) antibody, followed by incubation with a secondary antibody. Blots were detected with an Enhanced Chemiluminescence Assay Kit (Sigma), and the relative expression levels were quantified by Quantity One software (Bio-Rad).

### Measurement of inflammatory cytokines

The concentrations of IL-6 and tumor necrosis factor (TNF)-α in cell culture supernatants were analyzed strictly following the instructions provided by the manufacturer of the IL-6 (Catalog # EH2IL6, Thermo)/TNF-α (Catalog # KHC3012, Thermo) enzyme-linked immunosorbent assay (ELISA) Kit. Optical density was estimated using a Microplate Reader (Bio-Rad) at 450 nm, and the concentration of each sample was determined based on optical density and standard concentration.

### Malondialdehyde (MDA) content detection

The MDA content in the supernatant of the sonicated cells was assayed with the MDA Content Assay Kit (Catalog # D799762, Sangon biotech, Shanghai, China) following the manufacturer’s procedure.

### Sample collection

Normal cartilages (n = 28) and pathological cartilages (n = 28) were obtained from patients with femoral neck fractures without a history of OA or patients with end-stage OA during total hip replacement surgery at the Liyang People’s Hospital. OA severity was graded by the Kellgren–Lawrence (KL) grading scale. The 28 patients with OA were in stages I (n = 10), II (n = 8), III (n = 5) and IV (n = 5). Human cartilage samples were collected according to the protocol approved by the Ethics Committee of Liyang People’s Hospital, and all subjects signed a written informed consent form on the premise of understanding this study.

### RNA isolation and digestion

Isolation of total RNA from harvested cells and clinical samples was done with the miRNeasy Mini Kit (Catalog # 217,004, Shanghai, China). Agarose gel (1%) and NanoPhotometer^®^ spectrophotometer were used to assess the integrity and purity of total RNA. RNase R (3 U/μg, Geneseed, Guangzhou, China) was utilized for total RNA (2 μg) digestion at 37 °C for 15 min.

### Real-time polymerase quantitative chain reaction (RT-qPCR)

First-strand complementary DNA (cDNA) was synthesized using an M-MuLV cDNA Synthesis Kit (Sangon) or miRNA First Strand cDNA Synthesis (Sangon). Amplification reactions were carried out using a QuantiFast SYBR Green master mix (Qiagen) on LightCycler 480 equipment (Roche, Basel, Switzerland). Specific primers used were indicated in Table 1. Relative quantification was calculated with the 2^−ΔΔCt^ method. The internal references GAPDH and U6 were used for the analysis of gene expression.

**Table 1:** Specific primers utilized for RT-qPCR analysis

Name	Primers for qPCR (5′-3′)
circ_0020014	
Forward	CCCGTGTGAATGTGAAGAAA
Reverse	GGATGCATGGTAGGCACTTC
CTSB	
Forward	GCGCTGGGTGGATCTAGGA
Reverse	GTTGACCAGCTCATCCGACA
miR-24-3p	
Forward	GCGTGGCTCAGTTCAGCAG
Reverse	AGTGCAGGGTCCGAGGTATT
GAPDH	
Forward	GGAGCGAGATCCCTCCAAAAT
Reverse	GGCTGTTGTCATACTTCTCATGG
U6	
Forward	GCTTCGGCAGCACATATACTAA
Reverse	AACGCTTCACGAATTTGCGT

### RNA interference and cell transfection

A small interfering RNA (siRNA) targeting circ_0020014 (si-circ_0020014) and the non-targeting sequence (si-NC) were utilized to interfere with circ_0020014. The pcDNA-CTSB plasmid (CTSB) was used for overexpression of CTSB, with the empty pcDNA vector (Thermo) as a control. MiR-24-3p inhibitor (anti-miR-24-3p, 5′-CUGUUCCUGCUGAACUGAGCCA-3′) and mimic (miR-24-3p, 5′-UGGCUCAGUUCAGCAGGAACAG-3′) were respectively utilized to knock down and overexpress miR-24-3p, with anti-NC (5′-CGGUACGAUCGCGGCGGGAUAUC-3′) and miR-NC (5′-GGUUCGUACGUACACUGUUCA-3′) as controls. Transfection was carried out using the Lipofectamine RNAiMAX transfection reagent (Thermo).

### Dual-luciferase reporter assay

The wild-type (WT) fragments of WT-circ_0020014 and WT-CTSB 3′ UTR, as well as the mutational fragments MUT-circ_0020014 and MUT-CTSB 3′ UTR generated by the QuickChange II SiteDirected Mutagenesis Kit (Agilent Technologies, Beijing, China) were sub-cloned into the psiCHECK2 vector (Promega, Madison, WI, USA). CHON-001 cells were co-transfected with a constructed luciferase vector and miR-24-3p mimic or control mimic. Luciferase activities were measured with Dual-Luciferase Reporter Assay System (Promega).

### RNA-binding protein immunoprecipitation (RIP) assay

The Magna RIP RNA-Binding Protein Immunoprecipitation Kit (Catalog # 17-700, Sigma) was used for validation of the interaction between circ_0020014/CTSB and miR-24-3p. CHON-001 cells were lysed and co-incubated with magnetic beads conjugated with an antibody against IgG or Ago2. The abundance of circ_0020014, CTSB, and miR-24-3p in the precipitated RNA complex was analyzed by RT-qPCR.

### RNA pull-down assay

In short, biotinylated miR-24-3p mimic (bio-miR-24-3p) and control mimic (bio-miR-NC) (Sangon biotech) were transfected into CHON-001 cells. The cell lysates were incubated with streptavidin-coated magnetic beads (Thermo) at 4 °C. The abundance of circ_0020014/CTSB in the bead-captured RNA complex was detected by RT-qPCR.

### Statistical analysis

All values from at least three independent experiments were expressed as the means ± standard deviation. Data plotting was done with GraphPad Prism 8.0 (GraphPad, San Diego, CA). Statistical significance was determined by Student’s *t*-test for two groups or analysis of variance for three or more groups. Results were considered statistically significant for *P* < 0.05.

## Results

### IL-1β promoted chondrocyte apoptosis, inflammatory response and oxidative stress

**Fig. 1:**
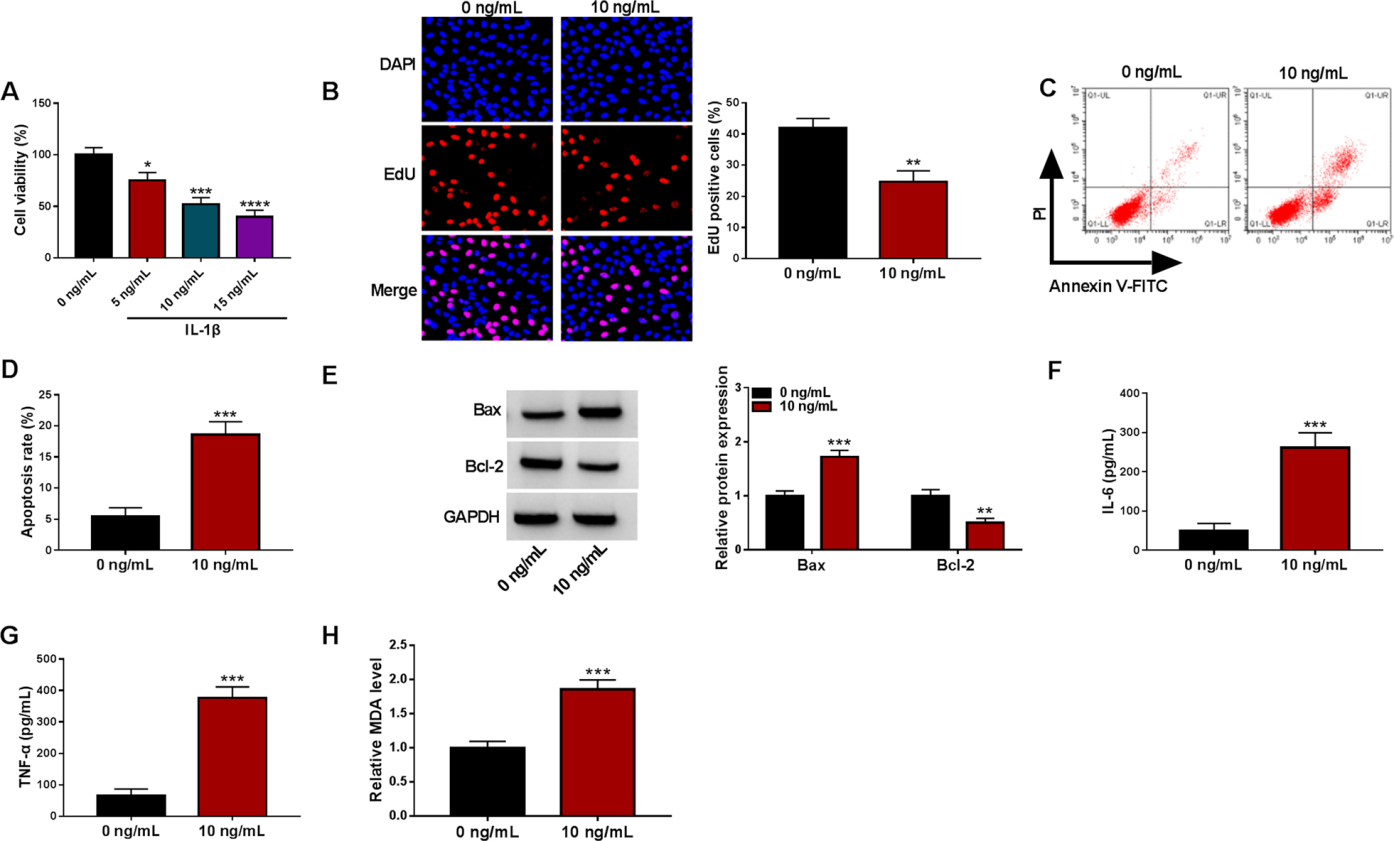
IL-1β promoted chondrocyte injury. **A** Cell viability in CHON-001 cells treated with different doses of IL-1β was detected by the MTT assay. **B**–**D** Cell proliferation and apoptosis in CHON-001 cells with or without IL-1β stimulation (10 ng/mL) were detected by the EdU and flow cytometry assays. **E** Relative protein levels of Bax and Bcl-2 in the above CHON-001 cells were evaluated by the WB analysis. **F**–**H** The levels of IL-6 and TNF-α released from the above cells and the production of MDA in these cells were analyzed with the respective kits. *P < 0.05, **P < 0.01, ***P < 0.001, and ****P < 0.0001

To investigate the toxicity effect of IL-1β on chondrocytes, different doses of IL-1β were used to stimulate CHON-001 cells for 24 h. Results of the MTT assay showed that IL-1β could cause a concentration-dependent decrease in the viability of CHON-001 cells, and 10 ng/mL of IL-1β was the half-inhibitory concentration (Fig. 1A). Subsequently, the effect of IL-1β (10 ng/mL) on chondrocytes was further explored. IL-1β stimulation repressed CHON-001 cell proliferation, as demonstrated by the EdU assay (Fig. 1B). Also, a marked increase in the apoptotic rate of CHON-001 cells was observed after IL-1β stimulation, as verified by the flow cytometry assay (Fig. 1C, D). As expected, CHON-001 cells exposed to IL-1β had high Bax protein levels and low Bcl-2 protein levels (Fig. 1E). In addition, IL-1β stimulation elevated the release of IL-6 and TNF-α from CHON-001 cells (Fig. 1F, G). Also, IL-1β stimulation caused the overproduction of MDA in CHON-001 cells (Fig. 1H). Collectively, these results implied a potential relationship between IL-1β and OA.

### Circ_0020014 was upregulated in OA patient-derived articular cartilages and IL-1β-stimulated chondrocytes

**Fig. 2:**
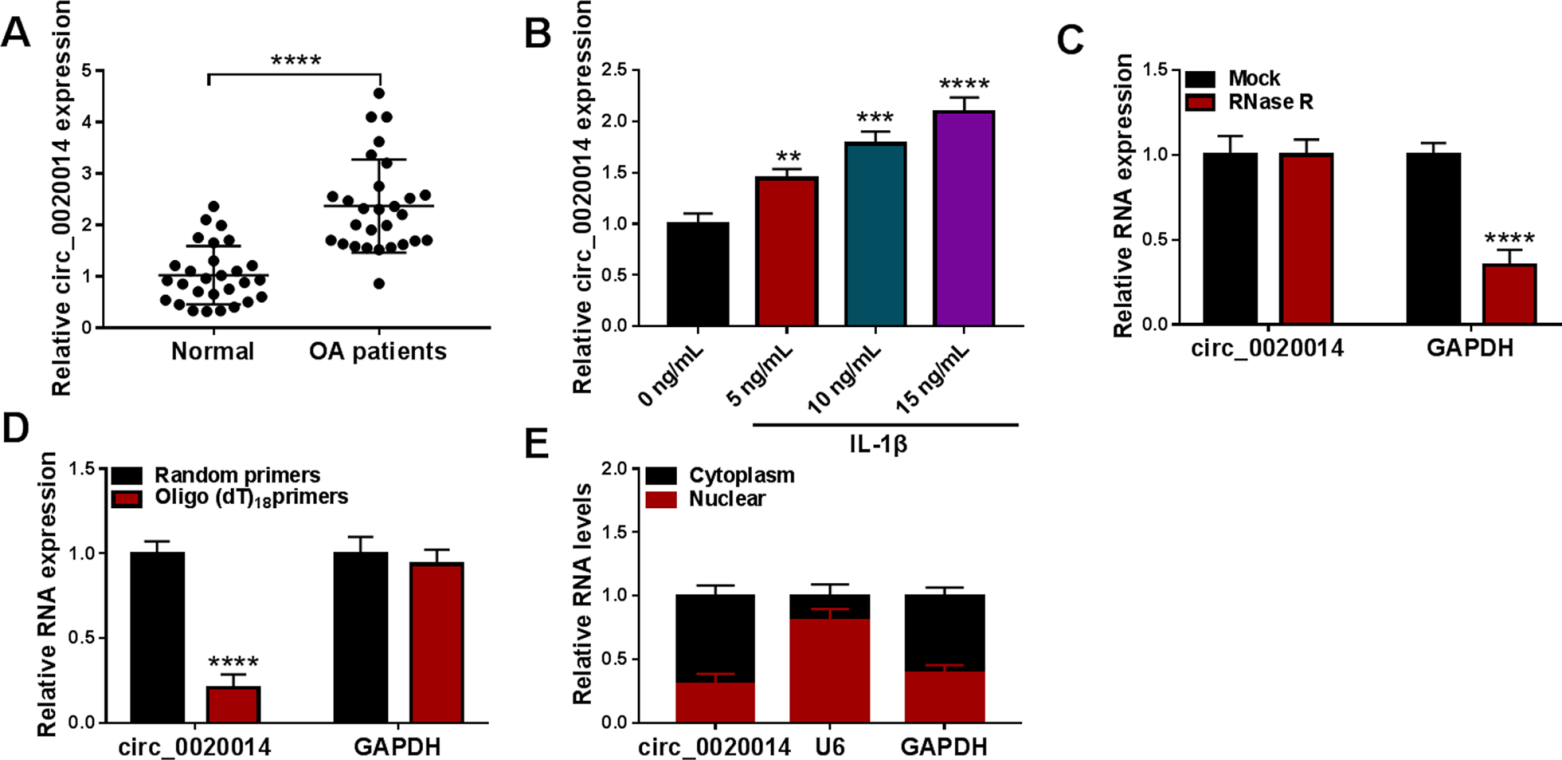
Circ_0020014 expression was increased in OA patient-derived articular cartilages and IL-1β-stimulated chondrocytes. **A** and **B** Relative expression of circ_0020014 in articular cartilages from OA patients and normal controls, as well as CHON-001 cells treated with different doses of IL-1β. **C** Relative expression levels of circ_0020014 and linear GAPDH in total RNA derived from CHON-001 cells with or without RNase R treatment. **D** Relative expression levels of circ_0020014 and linear GAPDH were analyzed by RT-qPCR using random or oligo(dT)_18_ primers in reverse transcription experiments. **E** The proportion of circ_0020014 in the cytoplasm and nucleus of CHON-001 cells. **P < 0.01, ***P < 0.001, and ****P < 0.0001

Normal articular cartilages and pathological articular cartilages were collected for further analysis. The safranine O-solid green staining showed that compared with normal articular cartilage, OA articular cartilage had a significant loss of proteoglycans, lighter staining of the cartilage matrix, severe calcification of the deep cartilage, and some areas showed discoloration (Additional file 1: Fig. S3). To understand the connection between circ_0020014 and OA pathogenesis, RT-qPCR was employed. The results showed a marked upregulation of circ_0020014 in OA patient-derived articular cartilages relative to normal control-derived articular cartilages (Fig. 2A). Also, IL-1β led to a concentration-dependent increase in the expression of circ_0020014 in CHON-001 cells (Fig. 2B). Expectedly, linear GAPDH could be degraded by RNase R, but circ_0020014 was more tolerant against RNase R (Fig. 2C). Random or oligo (dT)_18_ primers used in RT-qPCR showed that circ_0020014 expression was significantly reduced when the oligo (dT)_18_ primers were used, manifesting that circ_0020014 was a circRNA isoform without a poly-A tail (Fig. 2D). Subcellular fractions showed that circ_0020014 was predominantly located in the cytoplasm (Fig. 2E). The above results suggested that high circ_0020014 level might be related to OA pathogenesis.

### Circ_0020014 silencing lessened IL-1β-prompted chondrocyte injury

**Fig. 3:**
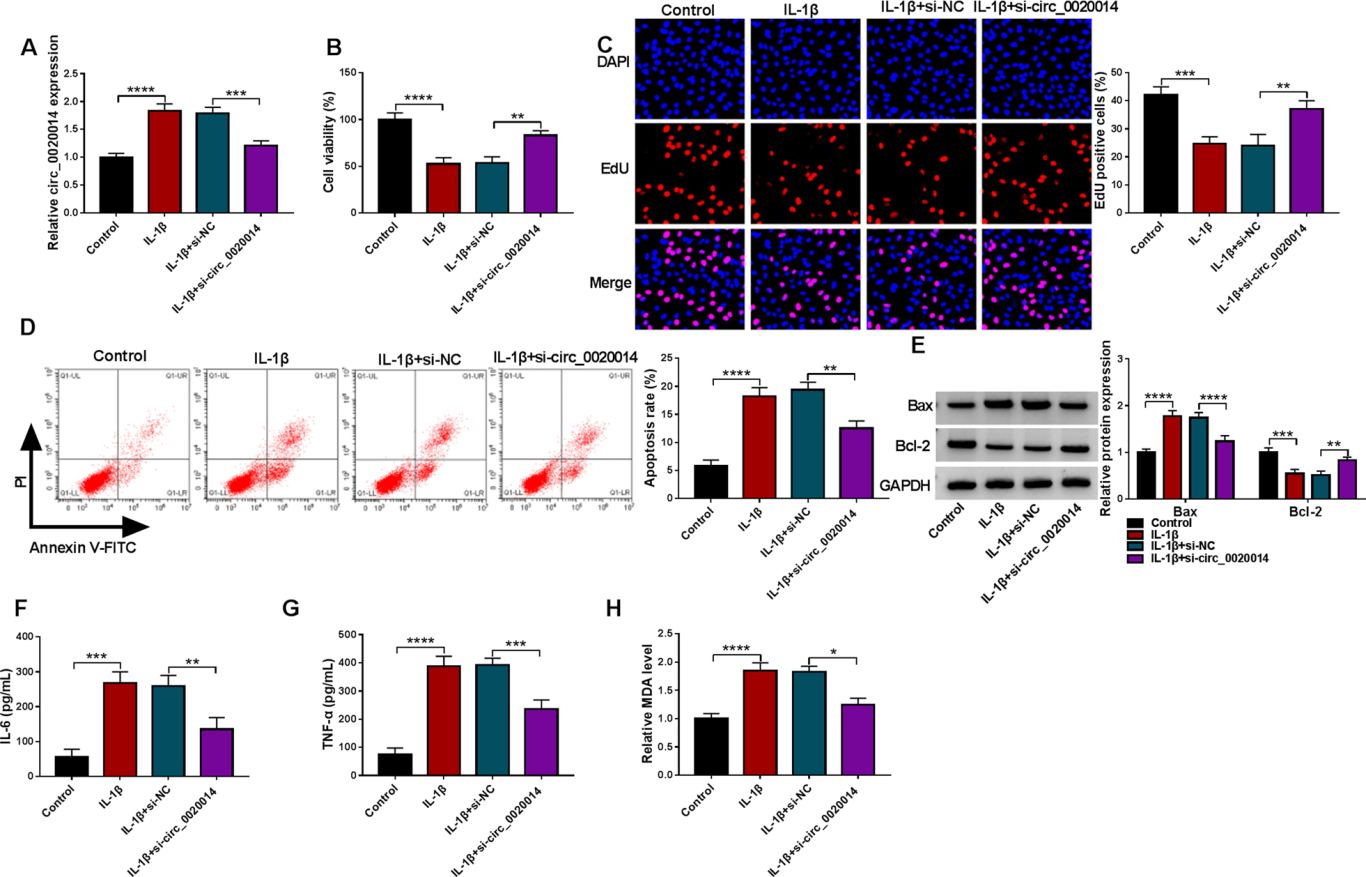
Circ_0020014 participated in IL-1β-prompted chondrocyte injury. **A** Relative expression of circ_0020014 in CHON-001 cells with or without IL-1β stimulation, as well as IL-1β-stimulated CHON-001 cells transfected with si-NC or si-circ_0020014. **B**–**D** Cell viability, proliferation, and apoptosis in the above CHON-001 cells were analyzed. **E** Relative protein levels of Bcl-2 and Bax in the above CHON-001 cells were detected. **F**–**H** The levels of IL-6, TNF-α, and MDA in the above CHON-001 cells were measured. *P < 0.05, **P < 0.01, ***P < 0.001, and ****P < 0.0001

The association of circ_0020014 with IL-1β-promoted chondrocyte injury was further explored by loss-of-function assays. IL-1β-mediated upregulation of circ_0020014 in CHON-001 cells was partially restored after circ_0020014 silencing (Fig. 3A). Moreover, IL-1β-mediated inhibition of cell viability and proliferation, as well as promotion of apoptosis, were attenuated after silencing of circ_0020014 (Fig. 3B–D). After circ_0020014 knockdown, upregulated Bax protein levels and downregulated Bcl-2 protein levels in CHON-001 cells forced by IL-1β stimulation were impaired (Fig. 3E). Also, the elevated levels of IL-6, TNF-α, and MDA urged by IL-1β treatment were lessened upon knockdown of circ_0020014 (Fig. 3F–H). Western blotting showed that IL-1β stimulation reduced collagen II protein levels and increased protein levels of MMP13 and ADAMTS5 in CHON-001 cells, but these changes caused by IL-1β were lowered after circ_0020014 silencing, suggesting that circ_0020014 could reduce the degeneration of CHON-001 cells (Additional file 1: Fig. S1). The above findings manifested that circ_0020014 participated in IL-1β-prompted chondrocyte injury.

### Circ_0020014 served as a miR-24-3p sponge

**Fig. 4:**
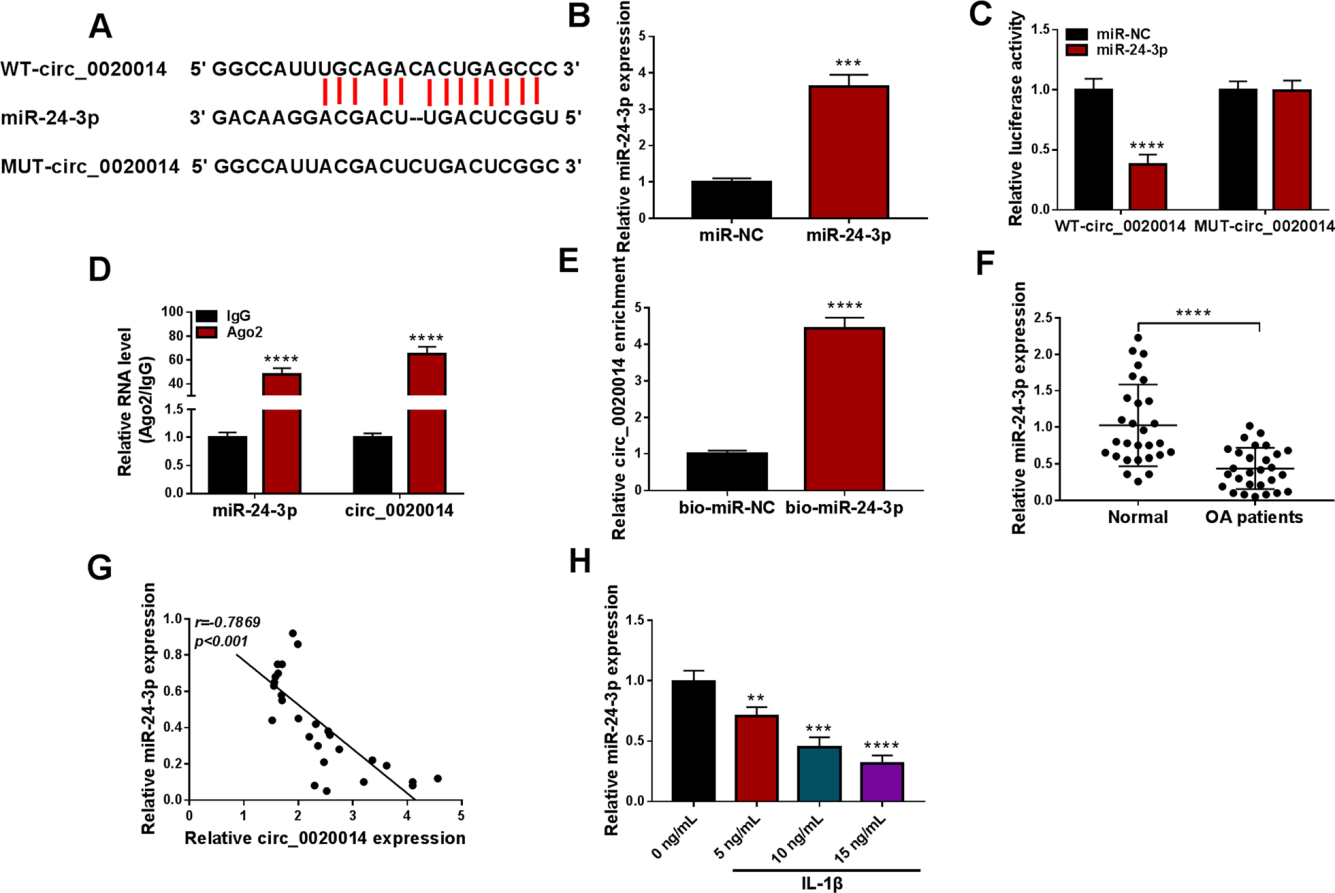
Circ_0020014 was verified as a miR-24-3p sponge. **A** Schematic illustration demonstrating complementary to the miR-24-3p seed sequence with circ_0020014. **B** RT-qPCR demonstrated the transfection efficiency of miR-24-3p mimic. **C** The relative luciferase activities were detected after co-transfection of WT-circ_0020014 or MUT-circ_0020014 and miR-24-3p mimic or miR-NC in CHON-001 cells. **D** RT-qPCR detection of the enrichment of circ_0020014 and miR-24-3p in the complex precipitated by the anti-Ago2 or anti-IgG antibody. **E** RT-qPCR evaluation of the abundance of circ_0020014 in the complex pulled down by the bio-miR-24-3p or bio-miR-NC probe. **F** Relative expression of miR-24-3p in articular cartilages from OA patients and normal controls. **G** Pearson’s correlation coefficient assessment of the correlation between circ_0020014 and miR-24-3p expression levels in articular cartilages from OA patients. **H** Relative expression of miR-24-3p in CHON-001 cells treated with different doses of IL-1β. **P < 0.01, ***P < 0.001, and ****P < 0.0001

Based on the result that circ_0020014 was preferentially localized in the cytoplasm of CHON-001 cells, we further explored the function of circ_0020014 as a miRNA sponge. Bioinformatics predicted that circ_0020014 might bind to miR-24-3p, and their possible binding sites were shown in Fig. 4A. To affirm their relationship, we employed miR-24-3p mimic to overexpress miR-24-3p in CHON-001 cells, as verified by RT-qPCR (Fig. 4B). Expectedly, miR-24-3p overexpression lowered the luciferase activity of the WT-circ_0020014 reporter, but it had no effect on the luciferase activity of the MUT-circ_0020014 reporter (Fig. 4C). The anti-Ago2 RIP assay showed that miR-24-3p and circ_0020014 could be enriched in the complex precipitated by the anti-Ago2 antibody (Fig. 4D). Also, the abundance of circ_0020014 was higher in the complex pulled down by the bio-miR-24-3p probe (Fig. 4E). Downregulation of miR-24-3p was detected in articular cartilages from OA patients (Fig. 4F and Additional file 1: Fig. S2), and miR-24-3p expression had a negative correlation with circ_0020014 expression (Fig. 4G). In addition, IL-1β resulted in a decrease in miR-24-3p expression in CHON-001 cells in a concentration-dependent manner (Fig. 4H). The above results manifested that circ_0020014 functioned as a miR-24-3p sponge.

### IL-1β prompted chondrocyte injury through the circ_0020014/miR-24-3p axis

**Fig. 5:**
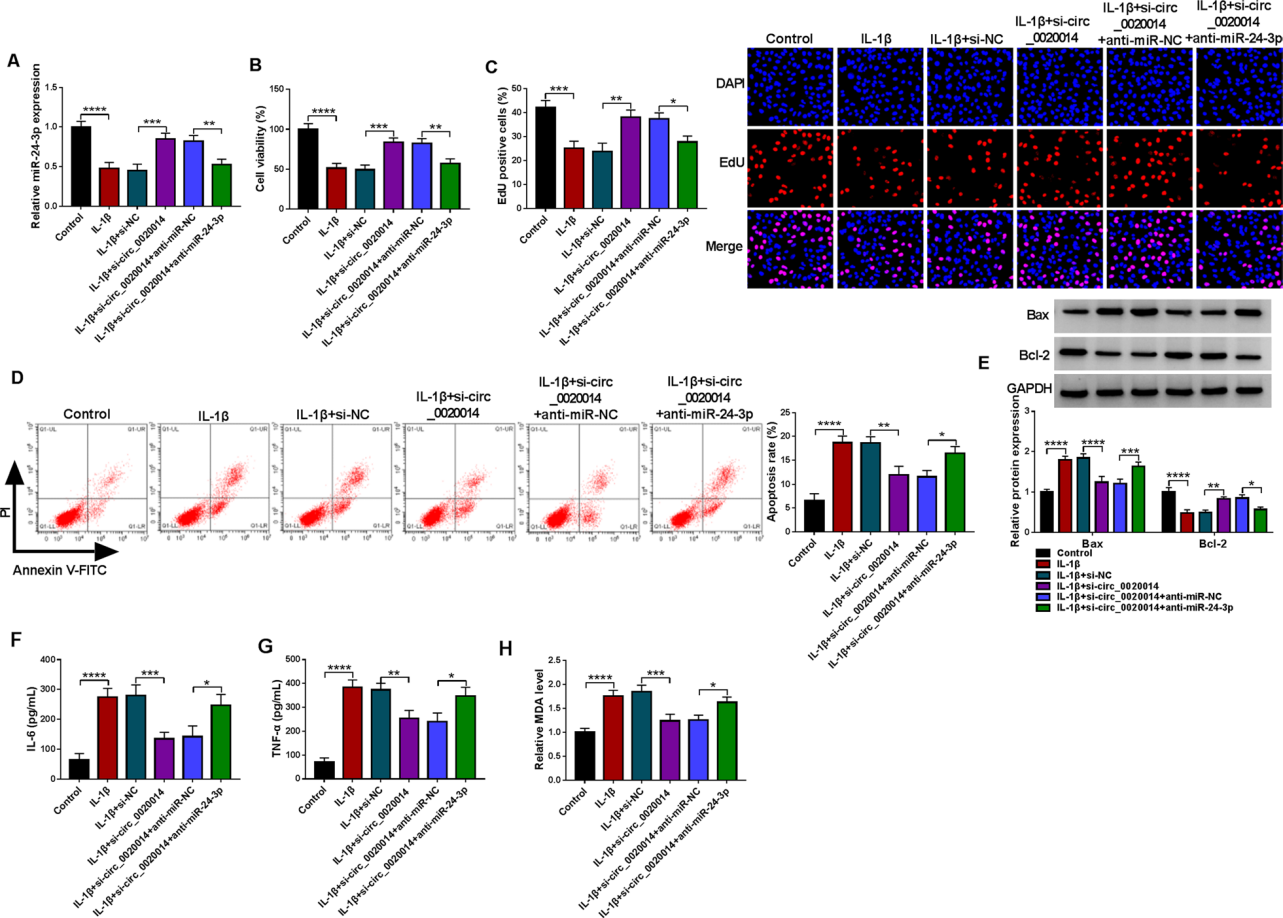
IL-1β induced chondrocyte injury via the circ_0020014/miR-24-3p axis. **A** RT-qPCR detection of miR-24-3p in CHON-001 cells with or without IL-1β stimulation, as well as IL-1β-stimulated CHON-001 cells transfected with si-NC, si-circ_0020014, si-circ_0020014+anti-miR-NC, or si-circ_0020014+anti-miR-24-3p. **B**–**D** Measurement of cell viability, proliferation, and apoptosis in the above-mentioned CHON-001 cells was done. **E** Analysis of Bcl-2 and Bax protein levels in the above-mentioned CHON-001 cells was done. **F**–**H** Detection of the levels of IL-6, TNF-α, and MDA in the above-mentioned CHON-001 cells was carried out. *P < 0.05, **P < 0.01, ***P < 0.001, and ****P < 0.0001

Whether IL-1β prompts chondrocyte injury through the circ_0020014/miR-24-3p axis was further investigated. IL-1β-induced downregulation of miR-24-3p in CHON-001 cells was attenuated by downregulation of circ_0020014, but the effect mediated by circ_0020014 knockdown was weakened by co-introduction of anti-miR-24-3p at the same time as circ_0020014 knockdown (Fig. 5A). Also, circ_0020014 downregulation lowered IL-1β-mediated repression of CHON-001 cell viability and proliferation, as well as promotion of CHON-001 cell apoptosis, but miR-24-3p silencing alleviated these effects contributed by circ_0020014 knockdown (Fig. 5B–D). Furthermore, circ_0020014 inhibition alleviated the changed levels of Bax and Bcl-2 in IL-1β urged by IL-1β stimulation, whereas co-silencing of miR-24-3p ameliorated these effects of circ_0020014 knockdown on these protein levels (Fig. 5E). In addition, miR-24-3p knockdown partially reversed the repressive effects of circ_0020014 silencing on IL-1β-induced elevation of IL-6, TNF-α, and MDA in CHON-001 cells (Fig. 5F–H). Collectively, these results manifested that circ_0020014 took part in IL-1β-prompted chondrocyte injury via interaction with miR-24-3p.

### CTSB acted as a miR-24-3p target

**Fig. 6:**
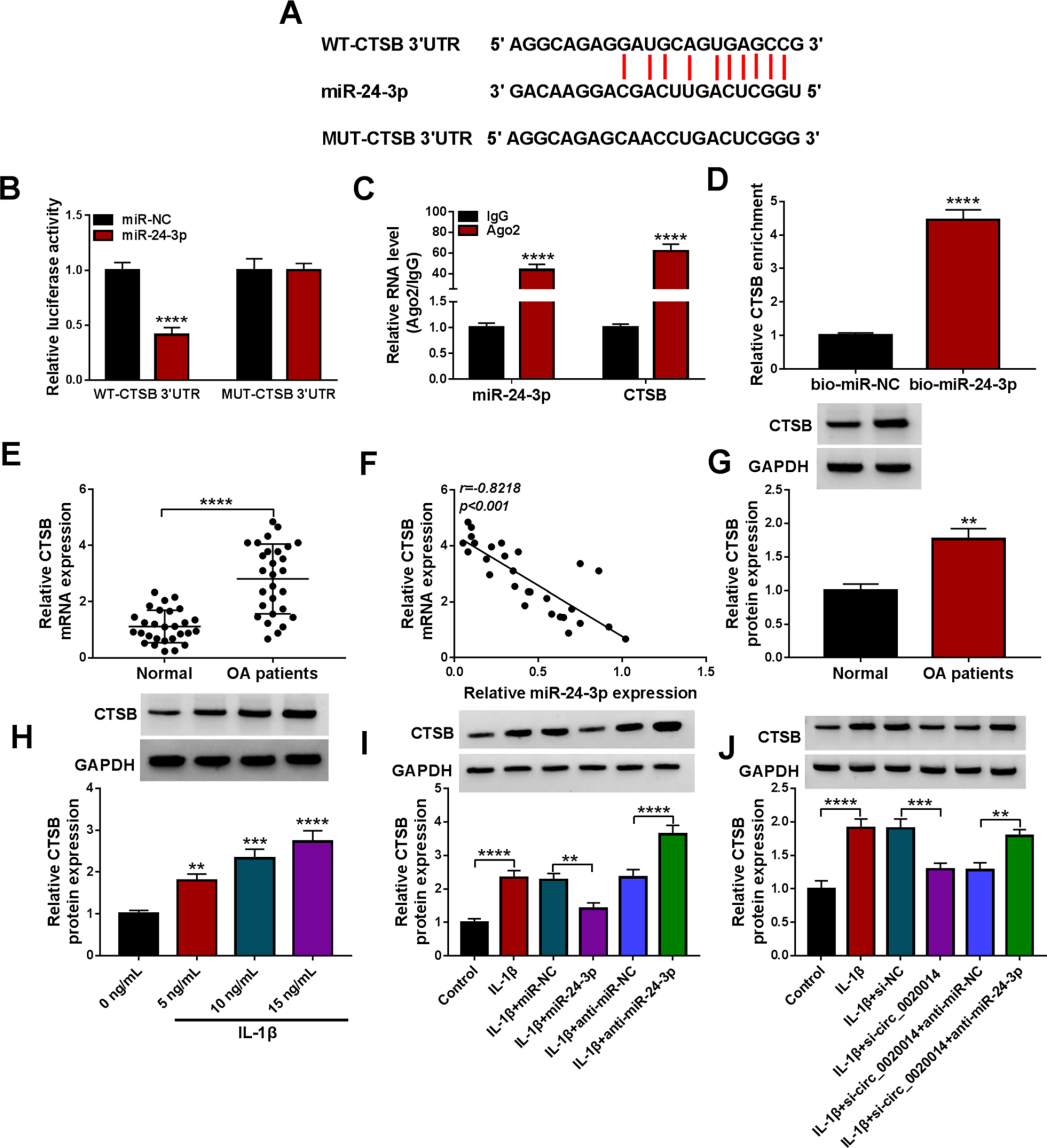
MiR-24-3p directly targeted CTSB. **A** Schematic representation of the complementarity of the 3’ UTR of CTSB to the seed sequence of miR-24-3p. **B**–**D** The binding of CTSB to miR-24-3p was validated by dual-luciferase reporter, RIP, and RNA pull-down assays. **E** RT-qPCR detection of the mRNA level of CTSB in articular cartilages from OA patients and normal controls. **F** Pearson’s correlation coefficient evaluation of the correlation between CTSB mRNA and miR-24-3p expression levels in OA patient-derived articular cartilages. **G** and **H** WB assessment of the protein level of CTSB in articular cartilages from OA patients and normal controls, as well as CHON-001 cells treated with different doses of IL-1β. **I** WB analysis of CTSB protein levels in CHON-001 cells with or without IL-1β stimulation, as well as IL-1β-stimulated CHON-001 cells transfected with miR-NC, miR-24-3p anti-miR-NC, or anti-miR-24-3p. **J** WB analysis of CTSB protein levels in CHON-001 cells with or without IL-1β stimulation, as well as IL-1β-stimulated CHON-001 cells transfected with si-NC, si-circ_0020014, si-circ_0020014+anti-miR-NC, or si-circ_0020014+anti-miR-24-3p. **P < 0.01, ***P < 0.001, and ****P < 0.0001

Next, we further probed into the downstream target of miR-24-3p. Among all the targets predicted by starbase, the cysteine protease CTSB which plays an important role in the regulation of metalloproteases, attracted our attention. The putative binding sites for miR-24-3p and the 3’UTR of CTSB are shown in Fig. 6A. Moreover, a reduction in the luciferase activity of the WT-CTSB 3′UTR reporter was obtained in the presence of miR-24-3p mimic (Fig. 6B). Also, the enrichment of miR-24-3p and CTSB mRNA was markedly higher in the anti-Ago2 group than in the control group (Fig. 6C). In addition, the mRNA of CTSB could be pulled down by the bio-miR-24-3p probe but not the control probe (Fig. 6D). We also observed that the mRNA level of CTSB was overexpressed in articular cartilages from OA patients (Fig. 6E), and its expression level had a negative correlation with miR-24-3p (Fig. 6F). Upregulated CTSB protein levels were obtained in OA patient-derived articular cartilages as well as IL-1β-stimulated CHON-001 cells (Fig. 6G, H). Overexpression of miR-24-3p weakened the elevated protein levels of CTSB in CHON-001 cells mediated by IL-1β stimulation, but miR-24-3p inhibitor enhanced the elevated protein levels of CTSB in IL-1β-stimulated cells (Fig. 6I). Furthermore, circ_0020014 inhibition lessened IL-1β-induced upregulation of CTSB, whereas miR-24-3p silencing ameliorated these effects mediated by circ_0020014 knockdown (Fig. 6J). The above results manifested that miR-24-3p directly targeted CTSB.

### MiR-24-3p participated in IL-1β-induced chondrocyte injury via targeting CTSB

**Fig. 7:**
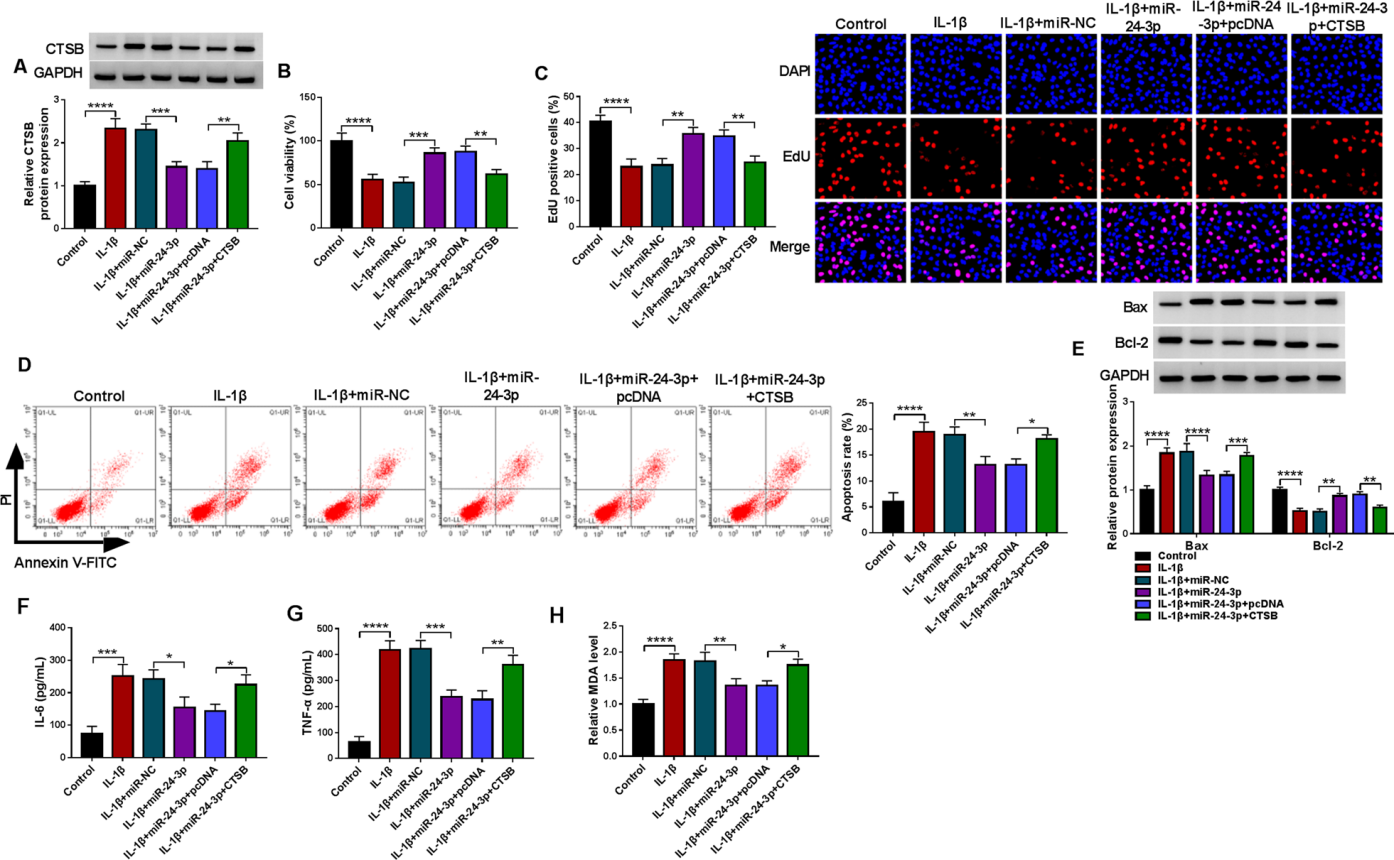
MiR-24-3p targeted CTSB to participate in IL-1β-induced chondrocyte injury. **A** Relative levels of CTSB protein in CHON-001 cells with or without IL-1β stimulation, as well as IL-1β-stimulated CHON-001 cells transfected with miR-NC, miR-24-3p, miR-24-3p+pcDNA, or miR-24-3p+CTSB. **B**–**D** Cell viability, proliferation, and apoptosis in the above-mentioned CHON-001 cells were determined. **E** Bcl-2 and Bax protein levels in the above-mentioned CHON-001 cells were assessed. **F**–**H** The production of IL-6, TNF-α, and MDA in the above-mentioned CHON-001 cells was measured. *P < 0.05, **P < 0.01, ***P < 0.001, and ****P < 0.0001

Subsequently, we further analyzed the relationship between miR-24-3p and CTSB in IL-1β-induced chondrocyte injury. The results showed that miR-24-3p overexpression alleviated IL-1β-induced upregulation of CTSB, whereas co-introduction of the CTSB overexpression plasmid attenuated this impact prompted by miR-24-3p overexpression (Fig. 7A). Also, elevated miR-24-3p expression attenuated IL-1β-mediated effects on CHON-001 cell viability, proliferation, and apoptosis, but these impacts contributed by miR-24-3p upregulation were impaired after CTSB overexpression (Fig. 7B–D). Furthermore, the changed levels of Bax and Bcl-2 in CHON-001 cells contributed by IL-1β stimulation were lowered after miR-24-3p overexpression, whereas co-introduction of CTSB ameliorated these effects mediated by miR-24-3p overexpression (Fig. 7E). Moreover, forced CTSB expression reversed the inhibitory effects of miR-24-3p overexpression on IL-1β-prompted overproduction of IL-6, TNF-α, and MDA in CHON-001 cells (Fig. 7F–H). The above results manifested that IL-1β prompted chondrocyte injury via the miR-24-3p/CTSB axis.

## Discussion

**Fig. 8:**
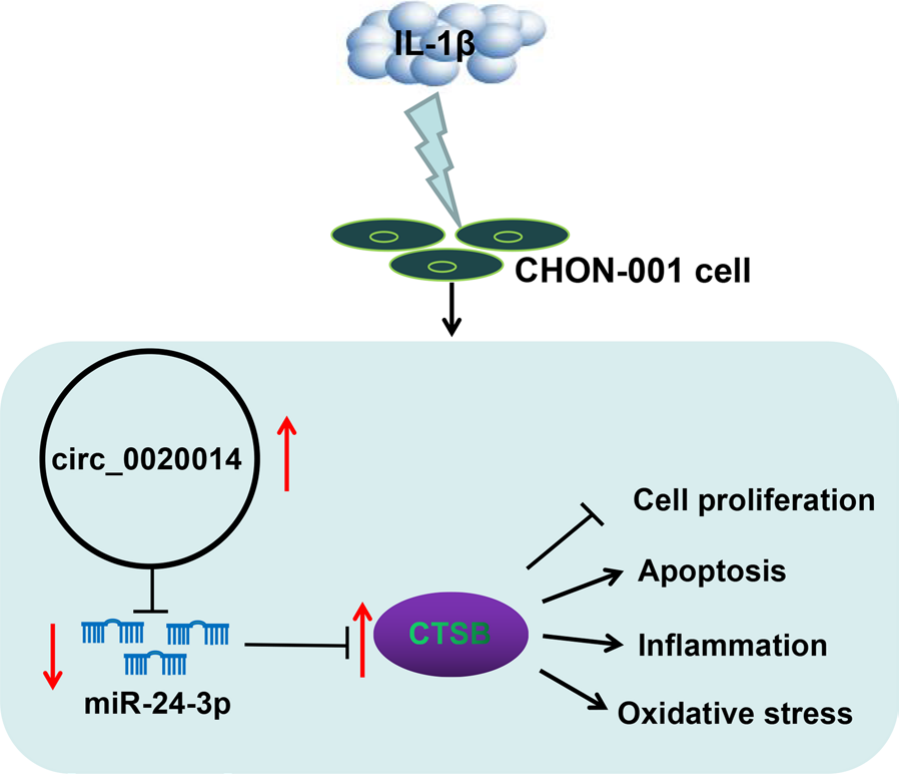
A schematic showing circ_0020014 involved in IL-1β-induced chondrocyte proliferation, apoptosis, inflammatory response, and oxidative stress by mediating the miR-24-3p/CTSB axis

In our current research, a new mechanism by which circ_0020014 participated in IL-1β-induced chondrocyte apoptosis, inflammatory response, and oxidative stress through mediating the interaction between miR-24-3p and the 3’UTR of CTSB was demonstrated, highlighting the involvement of circ_0020014 in the OA pathogenesis (Fig. 8).

Research by Wang et al. disclosed that circ_0020014 was differentially expressed in knee cartilage specimens between OA and Kashin-Beck disease, and peripheral blood circ_0020014 could discriminate between KBD and OA [25]. A recently published research showed that circ_0020014 took part in IL-1β-drove cell inflammation, apoptosis, and extracellular matrix degradation by the miR-613/ADAMTS5 axis in chondrocytes [29]. Our herein results suggested a significant elevation of circ_0020014 expression in OA patient-derived articular cartilages and IL-1β-stimulated chondrocytes. Moreover, circ_0020014 silencing relieved IL-1β-prompted chondrocyte proliferation repression, apoptosis, and inflammation, and these results were in line with the report by Yu et al. [29]. We also observed that circ_0020014 knockdown lowered IL-1β-prompted upregulation of MDA in chondrocytes, manifesting that circ_0020014 inhibition lessened IL-1β-prompted chondrocyte oxidative stress. Also, circ_0020014 silencing partly reversed the downregualtion of collagen II and the regulation of MMP13 and ADAMTS5 in CHON-001 cells induced by IL-1β stimulation, suggesting that circ_0020014 could reduce the degeneration of chondrocytes. All results highlighted that circ_0020014 was associated with IL-1β-prompted chondrocyte injury.

MiRNAs can regulate gene expression and play vital roles in human cells [8–10, 18]. Studies have reported that circRNAs act as molecular sponges for miRNAs through their miRNA-responsive elements, thereby releasing the repression of the target genes of the corresponding miRNAs [11]. For instance, circRNF20 mediated HIF-1α expression by sponging miR-487a in breast cancer [4]. Moreover, circZBTB44 sequestered miR-578 activity by acting as a miR-578 sponge in endothelial cells [30]. Recent evidence suggests that the circRNA-mediated endogenous RNA network plays a crucial role in OA [13]. The miRNA sponge function of circ_0020014 was demonstrated in the research. First, circ_0020014 was localized in the cytoplasm. Second, the possible interaction of circ_0020014 with miR-24-3p was predicted by bioinformatics. Third, circ_0020014 as a miR-24-3p molecular sponge was validated through a series of experiments (dual-luciferase reporter assays, RIP, RNA pull-down assays and RT-qPCR). Xu et al. exposed that the lowered expression of BCL2L12 mediated by miR-24-3p could attenuate IL-1β-induced chondrocyte injury [27]. Here, we observed that miR-24-3p expression was lowered in OA patient-derived articular cartilages and IL-1β-stimulated chondrocytes. Furthermore, downregulation of miR-24-3p lightened circ_0020014 silencing-mediated impacts on IL-1β-prompted chondrocyte proliferation repression, apoptosis, inflammation, and oxidative stress, manifesting that circ_0020014 could interact with miR-24-3p to take part in IL-1β-prompted chondrocyte injury. Whether circ_0020014 sponges other miRNAs involved in OA pathogenesis still needs to be explored.

A key factor in OA development is an increase in the catabolism of the extracellular matrix of chondrocytes [20]. Among all the targets of miR-24-3p predicted by bioinformatics, CTSB, which can degrade components of the extracellular matrix (such as proteoglycans and collagen) [3, 24], attracted our attention. CTSB could contribute to cartilage destruction in OA [32] and play a role in maintaining the chronic phase of OA [2]. Here, we identified CTSB as a miR-24-3p molecular target. Moreover, an apparent increase in the expression of CTSB was acquired in OA patient-derived articular cartilages and IL-1β-stimulated chondrocytes. Moreover, upregulated CTSB impaired the inhibiting impacts of miR-24-3p overexpression on IL-1β-prompted chondrocyte proliferation repression, apoptosis, inflammation, and oxidative stress, indicating that the miR-24-3p/CTSB axis mediated IL-1β-prompted chondrocyte proliferation repression, apoptosis, and inflammation. Importantly, circ_0020014 mediated CTSB expression via interaction with miR-24-3p in chondrocytes under IL-1β stimulation. This study identified miR-24-3p-mediated competing endogenous RNA crosstalk between circ_0020014 and CTSB in IL-1β-induced chondrocyte injury based on the competing endogenous RNA hypothesis. Further tasks need to be done to determine the role of circ_0020014 in OA progression through animal models. Bioinformatics and translational genomics have begun to elucidate the role and mode of action of functional peptides encoded by circRNA [26]. Whether circ_0020014 encodes small functional peptides will be further explored in the future.

In conclusion, we identified that the circ_0020014/miR-24-3p/CTSB competing endogenous RNA network participated in IL-1β-induced chondrocyte injury. The research offered the involvement of circ_0020014 in the OA pathogenesis.

### Supplementary Information

**Additional file 1**. **Figure S1**: Relative protein levels of collagen II, MMP13, and ADAMTS5 in CHON-001 cells with or without IL-1β stimulation, as well as IL-1β-stimulated CHON-001 cells transfected with si-NC or si-circ_0020014. *P < 0.05, **P < 0.01, and ****P < 0.0001.

**Additional file 2**. **Figure S2**: IHC staining showed miR-24-3p-positive cells in articular cartilages from OA patients and normal controls.

**Additional file 3**. **Figure S3**: The histomorphology of articular cartilage was observed by safranine O-solid green staining.

## Data Availability

The analyzed data sets generated during the present study are available from the corresponding author on reasonable request.
