# Supplementary figures and images for: Circ_0020014 mediates CTSB expression and participates in IL-1β-prompted chondrocyte injury via interacting with miR-24-3p
Source: J Orthop Surg Res. 2023 Nov 18;18:877. doi: 10.1186/s13018-023-04370-8 (PMC10657024; doi:10.1186/s13018-023-04370-8)

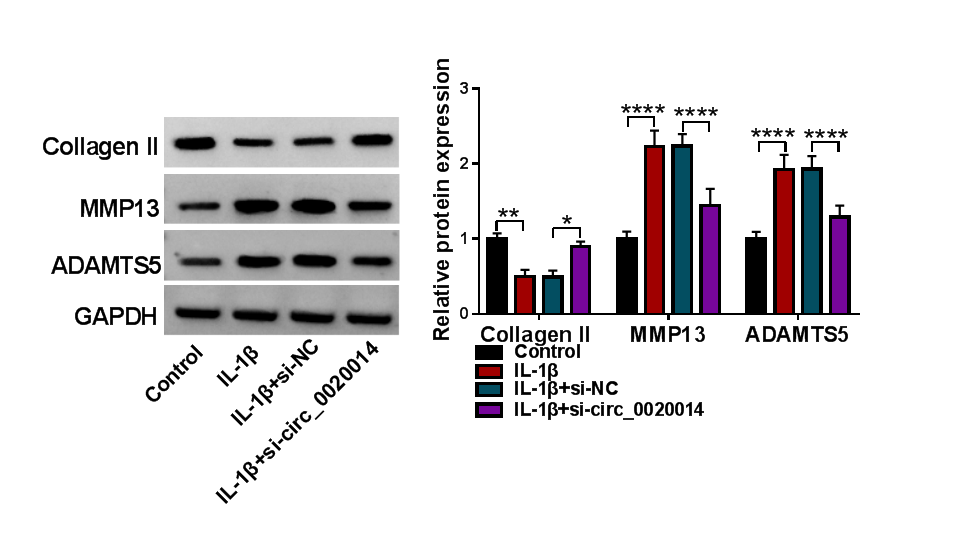

Supplement: Supplementary file 1 — Additional file 1. Figure S1: Relative protein levels of collagen II, MMP13, and ADAMTS5 in CHON-001 cells with or without IL-1β stimulation, as well as IL-1β-stimulated CHON-001 cells transfected with si-NC or si-circ_0020014. *P < 0.05, **P < 0.01, and ****P < 0.0001. [file 13018_2023_4370_MOESM1_ESM.tif]

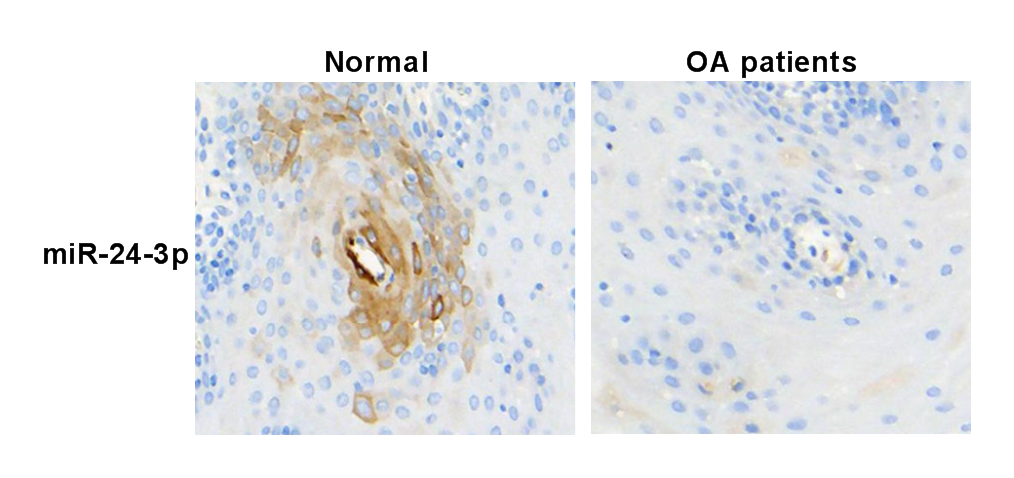

Supplement: Supplementary file 2 — Additional file 2. Figure S2: IHC staining showed miR-24-3p-positive cells in articular cartilages from OA patients and normal controls. [file 13018_2023_4370_MOESM2_ESM.tif]

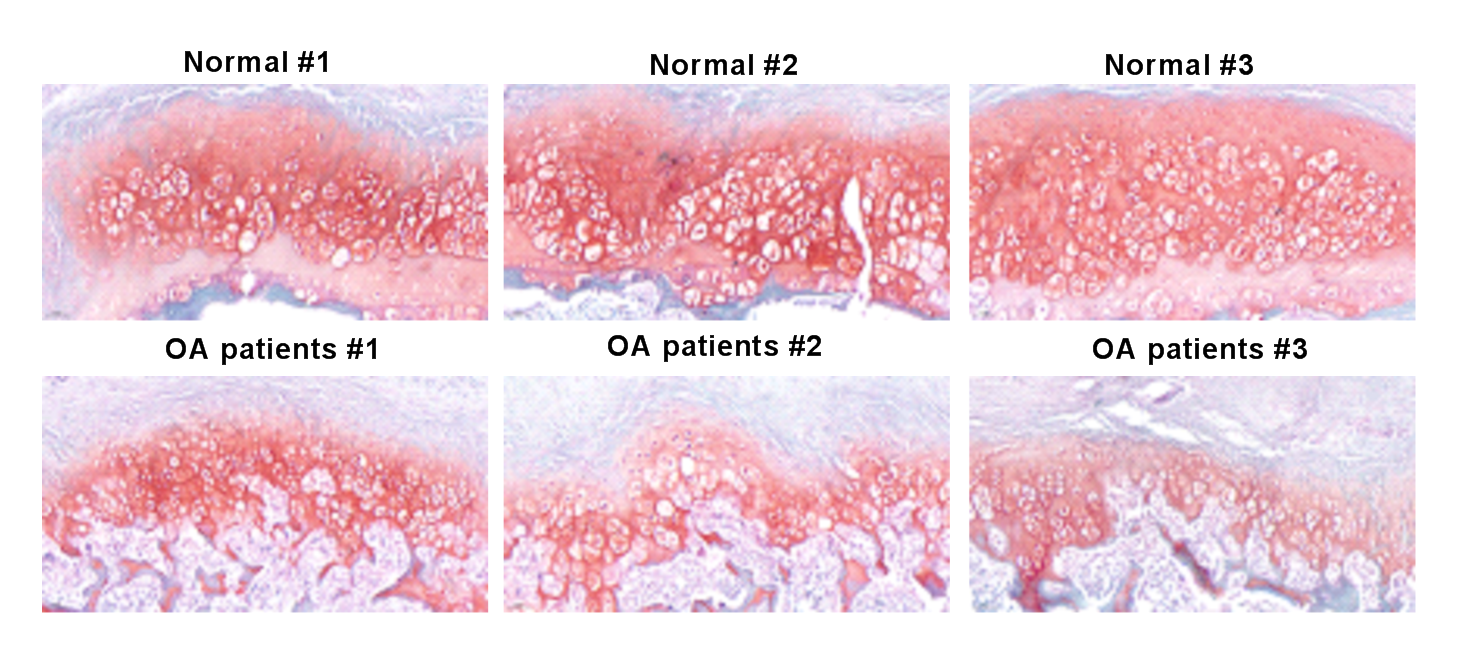

Supplement: Supplementary file 3 — Additional file 3. Figure S3: The histomorphology of articular cartilage was observed by safranine O-solid green staining. [file 13018_2023_4370_MOESM3_ESM.tif]
